# A cross-sectional study of low physical fitness, self-rated fitness and psychosocial factors in a sample of Finnish 18- to 64-year-old men

**DOI:** 10.1186/1471-2458-13-1113

**Published:** 2013-12-01

**Authors:** Karoliina S Kaasalainen, Kirsti Kasila, Jari Villberg, Jyrki Komulainen, Marita Poskiparta

**Affiliations:** 1 Faculty of Sport and Health Sciences, University of Jyväskylä, P.O.Box 35 (L) FI-40014, Jyväskylä, Finland; 2 Fit for Life Program, Viitaniementie 15a, FIN-40720, Jyväskylä, FIN-40720, Finland

**Keywords:** Physical fitness, Physical activity, Motivation, Psychosocial factors, Middle-aged, Men

## Abstract

**Background:**

The biological risk factors of inactivity and poor cardiorespiratory fitness are well established. However, risk groups are hard to reach and they may have misperceptions of their need for change. This study explored self-ratings of physical fitness (PF) and the relationship between objectively estimated physical fitness (PFI) and psychosocial factors among Finnish men of working-age.

**Methods:**

Cross-sectional data on 899 Finnish men (aged 18–64) were collected in 2011. Health- related physical fitness was evaluated with a physical fitness index calculated from the results of selected fitness tests. The men were subsequently classified into three groups: low, moderate and high PFI. Psychosocial factors and self-rated fitness were elicited in the questionnaire. The data were analysed with crosstabulations, chi square-test and logistic regression analysis.

**Results:**

One-fifth of the participants had low PFI. Forty-five per cent of the low-fit middle-aged (35–49 years) men self-reported poor PF, while 80 per cent of the younger (18–34 years) low-fit men self-reported moderate or good PF. The health benefits and recommended dose of physical activity were well known in all the PFI categories. The low-fit men were health conscious, but lacked adequate exercise skills, self-efficacy and social support. However, logistic regressions revealed that, in the younger men, likelihood of better knowledge was not related to higher PFI. Among the 50-to-64-year-old men, high PFI was not associated with a higher social support.

**Conclusions:**

Poor exercise skills, self-efficacy and social support were related to low PFI. Physical activity promotion for low-fit men should take into account age differences in the relationship between psychosocial factors and physical fitness. Thus, new and effective ways to establish social support and motivation for physical activity among low-fit men in all working-age groups are needed. Further research is also warranted on whether estimation of PFI could be used as a practical health counselling tool.

## Background

The health risks of inactivity, poor cardiorespiratory fitness and obesity are well established [1-4]. Only one-half of working-aged (18–64 years) men in Europe are sufficiently active [5] and up to 70 are overweight or obese [6]. Both poor cardiorespiratory fitness and abdominal obesity are associated with elevated risk for metabolic diseases and mortality among men [7]. Physical activity (PA) and a healthy diet are predictors of good physical fitness and favourable body composition. Health-related physical fitness (PF) describes an individual’s aerobic capacity, skeletal muscle strength and body composition [8]. The decreasing trend in PA and increasing obesity among working-aged men are public health issues [5]. Finnish studies have also expressed concern about poor cardiorespiratory fitness in young and working-aged men [9,10]. Although only 10 per cent of working-aged Finnish men have rated their PF as poor in population-based survey [11], the overall size of the low fitness population may be greater. People tend to overestimate their PA, while many studies have recruited participants from a motivated and physically active population [12,13].

To date, only a few physical activity interventions have been targeted at inactive men and most of these programmes have had poor reachability and only short-term effects [12,14]. Men are aware of the health benefits of adequate PA, but knowledge is a poor motivator for changes in health behaviour. Misperceptions of one’s own risk may be one explanation for the low interest in attending PA interventions. In previous studies up to 60 per cent of inactive people have overestimated their level of PA [13,15,16]. Physical fitness testing and individualized feedback have been used in health counselling in order to increase awareness of current PF and motivation for health behaviour changes [17-19]. However, comprehensive fitness tests have not improved counselling outcomes [17-19]. The results suggests that PF tests and feedback are not likely to contribute to the desired behaviour change process if individuals are already well aware of their current PF status or lack the confidence to implement the target behavioural change [18]. Hence, the reasons for low PF and inactivity do not reside in knowledge alone.

Most of people have the intention to be physically active, but less than a half of all attempts to maintain regular PA are successful [20]. Psychosocial factors, including knowledge, attitudes, intention, motivation social support and self-efficacy, affect PA adoption and maintenance [21,22]. Recent studies have concluded that although intention predicts PA, the latter can be also based on habitual and non-intentional behaviour [4,21,23-26]. A more evident moderator of behavioural change is self-efficacy, which is also included in most of the health behaviour theories [26]. Self-efficacy is a personal belief in one’s ability to engage in a desired behaviour [27]. The successful adoption of regular PA is argued to be influenced by an improvement in self-efficacy and reduction in the perceived barriers [22]. Barriers to PA can be environmental, and hence to some extent modifiable (e.g. social support, lack of exercise facilities), or individual and less likely to be modifiable (e.g. poor skills, lack of motivation) [28-30]. Modifiable and non-modifiable factors both have an influence on individuals’ enjoyment of physical activities and exercise. Genetics is a central non-modifiable factor determining PF, although PA history and health behaviour also have a considerable impact on PF [21].

Further understanding of the psychosocial factors that determine the adoption and maintenance of PA among low fitness men is needed. The aims in this study were to examine: 1) the relationship between self-rated physical fitness and objectively estimated physical fitness (PFI) and 2) the relationship between a low PFI and selected psychosocial factors.

## Methods

### Sample and study design

The study sample comprised 899 Finnish men who participated in a health promotion campaign, “The Adventures of Joe Finn”, during September 2011. The campaign was held in the market squares of 15 Finnish municipalities and was organized by the Fit for Life Program. The campaign offered all participants PF tests free of charge and personal feedback on the test results. The tests were conducted in a mobile test lab by trained personnel. Completion of all tests took about 15 minutes per participant. The inclusion criteria in to the study were gender (male), age (18–64) and completion both fitness tests and the health behaviour questionnaire. Participation in the study was voluntary and all participants gave their written consent. The study was approved by the University of Jyväskylä Ethical Committee.

### Physical fitness index

The fitness tests included hand grip strength (Saehan dynamometer), the Polar Fitness Test (Polar Electro, Kempele, Finland) and a body composition analysis (InBody 720- analyser). To our knowledge, this is the first time that this combination of fitness tests has been used to evaluate health-related PF. These fitness tests were chosen, as the aim of the campaign was to encourage sedentary men to participate in the tests and help them to become familiar with their PF. Exercise tests demanding strenuous physical effort were not deemed suitable for health counselling purposes. Previous studies suggest that hand grip-strength, Polar Fitness Test and body composition analysis by bioelectrical impedance (BIA) are feasible tests for population-based studies and the results correlate strongly with other assessment methods [1,31].

Hand grip strength describes the general fitness of the skeletal muscles and also predicts functional ability and risk for chronic diseases in the last years of life [32]. Hand grip strength was measured with a Saehan dynamometer and the results were compared with age and gender specific reference values. The references were based on fitness test data on working-aged Finnish men collected by the LIKES Research Center for Sport and Health Sciences between the years 2007-2011 [33].

Aerobic fitness was measured with the Polar Fitness Test, which predicts a person’s aerobic capacity (VO_2_max) [34]. Estimation of VO_2_max was based on resting heart rate, heart rate variability, gender, age, height, body weight and self-reported level of long-term physical activity. The resulting value was compared to international reference standards of aerobic capacity for gender and age group, and the individual was subsequently assigned to one of the fitness categories, which ranged from 1 to 7 [35]. Although VO_2_max was assessed indirectly, the Polar Fitness Test has been shown to be a reliable test of aerobic fitness in population-based studies [36]. The mean error between the Polar Fitness Test and laboratory-measured maximal oxygen uptake has varied between 6.5 and 8.2 per cent [37].

Body composition was measured by using an InBody 720 analyser. The body composition analysis estimates body weight, percentage of total body fat (fat%), visceral fat area (VFA) (cm^2^) and skeletal muscle mass (SMM) (kg/m). VFA describes abdominal obesity, which has been associated with increased risk for mortality and metabolic diseases [2,7]. Obese individuals with good PF have had less internal fat than obese and unfit individuals [1,38]. SSM describes fat-free mass, which has positive associations with functional ability and energy metabolism [39]. In comparison to the other body composition assessment methods (e.g. DEXA and MRI), the BIA has reasonable validity [31].

The final physical fitness index (PFI) described health-related fitness with a numeric scale. The PFI was computed from the results of the following fitness test variables: estimated aerobic capacity (VO_2_max), hang grip strength (kg/kg), percentage of total body fat (fat%), SSM (kg/m) and VFA (cm^2^). All the test results were converted to standardized points and then weighted with the following equations: Aerobic fitness (VO_2_max), points = 0,5 × [10 × (ml/kg/min - (-0,2835 × age + 50,307)) / 30], body fat (%), points = 0,1 × [ - (10 × fat% - (0,143 × age + 15,264))/ 24], VFA (cm^2^), points = 0,15 × [ - (10 × (cm2 - (1,326 × age + 56,031))) / 140], Hand grip strength (kg/kg), points = 0,15 × [10 × (kg/kg – (-0,036 × age + 22,33)) / 10], SSM (kg/m), points = 0,1 × [10 × (kg/m - (-0,0037 × age + 0,83)) / 0,5]. The final PFI ranges from ′-5, +5′, where < -3 = very poor, < -1 = poor, < +1 = acceptable, < +3 = good and > +3 = very good PF. For the statistical analyses, the PFI was recoded into low PF (PFI ≤ -1), moderate PF (PFI <1) and high PFI (PFI ≥ 1) classes.

### Self-rated physical fitness and sufficiency of physical activity

Self-rated PF was elicited with the question “What do you think about your current level of physical fitness?” The response alternatives were given on a 5-point scale (1 = very good…5 = very poor). For the statistical analyses, self-rated PF was recoded into three classes (good, moderate and poor). Furthermore, perceived sufficiency of PA was elicited with the statement “I am sufficiently physically active”. The response alternatives were given on a 5-point scale (1 = totally agree…4 = totally disagree, 5 = I don’t know). The responses were assigned to one of three classes (1 = agree, 2 = disagree and 0 = I don’t know). The categories were dichotomised for the statistical analyses into two classes (1 = agree, 2 = disagree or I don’t know).

### Health, physical activity and readiness for physical activity change

General health status was elicited in the questionnaire by reference to a list of chronic diseases [11]. Participants self-evaluated their level of PA, which was later assigned to one of 4 categories (1 = over 5 h/week (wk), 2 =3-5 h/wk, 3 = 1-3 h/wk and 4 = 1 or 0 h/wk). The classification was constructed on the basis of the answers given on the Polar Fitness Test background form. The PA assessment included descriptions of the frequency, duration and intensity of PA, including both conditioning and non-conditioning PA. Commuting activity to work and frequency of gym/strength training were also elicited in the health behaviour questionnaire. Readiness for PA change was elicited with the question “Have you increased your level of PA during the past year?” (1 = No, and I have no intention to increase it, 2 = No, but I intend to increase it in the near future, 3 = I have tried to increase it, 4 = I have increased it considerably, and 5 = I have been physically active on regular basis”).

### Psychosocial factors

The health behaviour questionnaire included 19 statements (Cronbach’s alfa 0.90) on psychosocial factors (Table 1). The items concerned knowledge on the health benefits of PA, perceived PA skills, goal setting, social support and self-efficacy. The psychosocial items were selected on the basis of previous studies [40-43]. Participants were asked to assess how well the statements matched their situation. The original response alternatives were given on a 5-point scale (1 = totally agree, 2 = somewhat agree, 3 = somewhat disagree, 4 = totally disagree 5 = I don’t know). The responses were subsequently assigned to three classes (1 = agree, 2 = disagree and 0 = I don’t know). The categories were dichotomised for the statistical analyses into two classes (1 = agree, 2 = disagree or I do not know). In the further analysis, the psychosocial items were divided into five sub-dimensions (knowledge, skills, goal setting, social support and self-efficacy). Those who agreed with all the score-related items formed the high-score group.

**Table 1:** Scale of psychosocial factors and associations with physical fitness index

	**Low (n = 172)**	**Moderate (n = 386)**	**High (n = 341)**	**p**^**1**^
	**(%)**	**(%)**	**(%)**	
**Knowledge (Chronbach’s** α **= 0.80)**				
I know health benefits of PA	95	98	97	0.201
I know how often I should exercise	90	94	95	0.165
I know how many hours in a week I should exercise	85	86	90	0.115
I know the intensity at which I should exercise	76	77	83	0.042
I know where I can get social support for exercise	59	72	77	<0.001
**Skills (Chronbach’s** α **= 0.71)**				
I have sought information on exercise	52	63	75	<0.001
I can seek exercise alternatives	75	85	90	<0.001
I have found an agreeable way to exercise	67	85	95	<0.001
I have good exercise skills	63	82	93	<0.001
**Goal setting (Chronbach’s** α **= 0.76)**				
I have set goals for exercise	50	64	78	<0.001
I can achieve my exercise goals	61	77	86	<0.001
**Self-efficacy ( Chronbach’s** α **= 0.80)**				
I am able to exercise when I am tired	51	55	76	<0.001
I am able to exercise when I am bad tempered	62	78	86	<0.001
I am able to exercise when I am busy	38	52	67	<0.001
I am able to exercise although people close to me do not regard highly PA	71	87	90	<0.001
I am able to restart exercise after an inactive period	80	88	95	<0.001
**Social support (Chronbach’s** α **=0.75)**				
People close to me support my PA	70	82	88	<0.001
People close to me have a high regard for PA	75	87	90	<0.001
I believe that by being active I can contribute to PA by people close to me	75	82	88	0.001

p^1^ = significance tested by chi square-test.

### Statistical analyses

Data were analysed using SPSS for Windows 18.0. Basic descriptive data frequencies and cross-tabulation with chi square-test were calculated for demographics (education, marital status, employment, age, BMI, chronic diseases), physical activity (total PA level, gym training and commuting activity) and the psychosocial variables. Bivariate and multivariate logistic regressions were used to test associations between the psychosocial variables and PFI in three age categories (18–34, 35–49 and 50–64). Main effects of the psychosocial variables were analysed in logistic regression analyses. The psychosocial variables were entered in the models first individually and subsequently by the stepwise method. Only statistically significant results of the stepwise models were reported. The results of the logistic regression analyses are presented as odds ratios (OR) and 95% confidence intervals (CI). Low-fit men were used as a reference group in all models.

## Results

The respondents’ (N = 899) mean age was 43.9 (SD = 12.7). The majority of the study participants were employed (69%) and married or cohabiting (66%). Nineteen per cent of the men were located in the low-fitness class (low-fit), 42 per cent in the moderate fitness class (mod-fit) and 38 per cent in the high fitness class (high-fit). The low-fit men were less educated and they reported more diseases than the high-fit men. Almost all the low-fit men were overweight or obese, and one-fifth engaged in physical activities at least three hours per week (Table 2). Also among the low-fit men, one-fifth reported that they had either increased PA during the past year or were permanently active.

**Table 2:** Associations between Physical Fitness Index, demographics and Physical Activity behaviour

	**Low PFI (N = 172)**	**Moderate PFI (N = 386)**	**High PFI (N = 341)**	**Total (N = 899)**	**p**^**1**^
	**f (%)**	**f (%)**	**f (%)**	**f (%)**	**(Χ**^**2**^**)**
**Age**					
18-34	48 (27.9)	103 (26.7)	106 (31.1)	257 (28.6)	ns
35-49	65 (37.8)	140 (36.3)	120 (35.2)	325 (36.2)	
50-64	59 (34.3)	143 (37.0)	115 (33.7)	325 (36.2)	
**Education**					
Low (-9 year)	16 (9.8)	37 (10.2)	29 (8.8)	82 (9.6)	0.003
Medium (9–12 year)	88 (53.7)	202 (56.0)	140 (42.9)	430 (50.5)	(16.4)
High (12- year)	60 (36.6)	123 (33.8)	88 (48.3)	213 (39.9)	
**Chronic diseases**					
No or not reported	129 (75.0)	309 (80.9)	292 (85.9)	731(81.8)	0.009
at least one	43 (25.0)	73 (18.9)	48 (14.1)	164(18.2)	(9.4)
**BMI**					
<25	14 (8.1)	130 (33.8)	231 (67.7)	375 (41.8)	<0.001
25-29,9	60 (34.9)	215 (57.2)	109 (32.0)	386 (43.0)	(389.5)
≥30	98 (57.0)	38 (9.9)	1 (0.3)	137 (15.3)	
**Activity level**					
<1 h/week	70 (40.7)	68 (17.6)	1 (0.3)	139 (15.6)	<0.001
1-3 h/week	71 (41.3)	224 (58.0)	115 (33.7)	410 (45.4)	(251.6)
>3 h/week	31 (18.0)	94 (24.4)	225 (66.0)	350 (38.9)	
**Frequency of strength training**					
at least 3 times/wk	32 (18.5)	116 (30.4)	146 (42.9)	294 (32.8)	<0.001
1-2 times/wk	55 (31.6)	139 (36.4)	129 (37.9)	323 (36.1)	(45.4)
less than once a week	86 (49.7)	127 (33.2)	65 (19.1)	278 (31.1)	
**Commuting to work by walking or cycling/ week**					
≤ 1 h /wk	143 (83.1)	284 (73.6)	192 (56.3)	619 (68.9)	<0.001
> 1 h/wk	29 (16.9)	102 (26.4)	149 (43.7)	280 (31.1)	(44.7)
**Motivational readiness**					
No intention to increase PA	8 (4.7)	42 (11.0)	58 (17.2)	108 (12.1)	<0.001
Intention to increase PA	56 (32.6)	91 (23.9)	41 (12.1)	188 (21.1)	(114.1)
Tried to increase PA	75 (43.6)	146 (38.3)	89 (26.3)	310 (34.8)	
Increased PA during the past year	30 (17.4)	69 (18.1)	62 (18.3)	161 (18.1)	
PA on regular basis	3 (1.7)	33 (8.7)	88 (26.0)	124 (13.9)	

F = frequencies,% = percentage, p^1^ = significance tested by chi square-test, Χ^2^ = chi square, ns = no significant.

When self-reported and measured PFI were compared in the different age-groups, poor PF was most frequently reported by the middle-aged men. Almost 80 per cent of the low-fit men in the youngest group self-estimated moderate or good PF. The youngest low-fit men also the most often reported engaging sufficiently in PA (Table 3). One-third (29%) of the youngest low-fit men self-reported less than one hour of PA per week, while among the middle-aged and oldest group the corresponding percentages were 50 and 41.

**Table 3:** Percentages of Self-rated physical fitness and sufficient physical activity in different PFI-categories and age groups

	**Low (n = 172)**	**Moderate (n = 386)**	**High (n = 341)**	**p**
**Self-rated PF**	**f (%)**	**f (%)**	**f (%)**	**(Χ**^**2**^**)**
Age 18-34				
good	6 (12.5)	45 (43.7)	81 (76.4)	<0.001
moderate	31 (64.6)	47 (45.6)	22 (20.8)	60.4
poor	11 (22.9)	11 (10.7)	3 (2.8)	
Age 35-49				
good	3 (4.6)	29 (20.7)	86 (71.7)	<0.001
moderate	33 (50.8)	97 (69.3)	33 (27.5)	153.2
poor	29 (44.6)	14 (10.0)	1 (0.8)	
Age 50-64				
good	8 (13.6)	38 (26.6)	76 (66.1)	<0.001
moderate	28 (47.5)	89 (62.2)	37 (32.2)	91.9
poor	23 (39.0)	16 (11.2)	2 (1.7)	
**I engage sufficiently in PA**				
18-34	18 (37.5)	56 (54.4)	84 (79.2)	<0.001
35-49	13 (20.0)	64 (45.7)	98 (81.7)	<0.001
50-64	11 (18.6)	71 (49.7)	98 (85.2)	<0.001

F = frequencies,% = percentage, p = significance tested by chi square-test, Χ^2^ = chi square.

### Psychosocial factors and physical fitness

The health benefits and recommended dose of PA were well known. However, 40 per cent of the low-fit men did not know where to obtain social support (Table 1). Only half of the low-fit men reported seeking information on exercise or setting exercise goals. The low-fit men were also less confident than mod- and high-fit men of their ability to be physically active in different life situations.

The results of the logistic regression analyses revealed several age-specific differences in the odd ratios for high scores in the psychosocial variables (Table 4). In both logistic regression models, the results showed that, in the youngest group, the moderate-fit men were more likely to have a higher knowledge score than the low-fit men. Although the youngest moderate-fit men did not report better skills than their low-fit peers, among the middle-aged and the oldest groups the likelihood of having good skills was higher in the moderate and high PFI classes. Also in the stepwise model, the ORs for a high score in skills across ages 35–64 remained statistically significant between the low and high-fit men.

**Table 4:** Odds ratios (OR) for selected psychosocial factors across different fitness categories and age groups

	**Low**	**Moderate (model 1)**	**Moderate (model 2)**	**High (model 1)**	**High (model 2)**
	**OR (95% CI)**	**OR (95% CI)**	**OR (95% CI)**	**OR (95% CI)**	**OR (95% CI)**
**Knowledge**					
18-34	1.00	2.84 (1.40-5.76)*	2.59 (1.25-5.36)*	1.84 (0.92-3.69)	ns
35-49	1.00	1.52 (0.84-2.74)	ns	2.58 (1.39-4.80)*	ns
50-64	1.00	1.66 (0.90-3.05)	ns	3.07 (1.58-5.95)*	ns
**Skills**					
18-34	1.00	1.95 (0.98-3.91)	ns	2.72 (1.35-5.49)*	ns
35-49	1.00	2.19 (1.17-4.07)*	ns	7.39 (83.36-14.53)**	5.35 (2.34-10.85)**
50-64	1.00	2.49 (1.23-5.00)*	2.49 (1.23-5.00)*	6.15 (2.98-12.68)**	6.15 (2.98-12.68)**
**Goal setting**					
18-34	1.00	2.51 (1.40-5.06)*	ns	3.46 (1.69-7.08)*	ns
35-49	1.00	1.48 (0.82-2.68)	ns	4.55 (2.37-8.73)**	ns
50-64	1.00	1.66 (0.90-3.06)	ns	3.44 (1.78-6.65)**	ns
**Self-efficacy**					
18-34	1.00	1.52 (0.73-3.10)	ns	3.22 (1.56-6.64)**	2.36 (1.10-5.09)*
35-49	1.00	1.97 (1.01-3.85)*	ns	3.83 (2.44-9.56)**	2.90 (1.40-6.00)**
50-64	1.00	2.48 (1.15-5.31)*	ns	4.04 (1.87-8.76)*	ns
**Social support**					
18-34	1.00	2.51 (1.22-5.15)*	2.22 (1.04-4.74)*	3.64 (1.72-7.69)*	2.74 (1.25-5.98)*
35-49	1.00	1.86 (1.02-3.37)*	ns	3.55 (1.86-6.79)**	ns
50-64	1.00	1.70 (0.80-3.23)	ns	1.90 (0.97-3.71)	ns

*p < 0.05, **p < 0.001, OR = odds ratio, CI = confidence interval, ns = no significant, Model 1 = ORs for single psychosocial scores, Model 2 = stepwise model for psychosocial scores.

The moderate-fit younger men scored higher in goal setting than their youngest low-fit counterparts, while among the middle-aged and older men only the high-fit were likely to have a high goal-score. In the stepwise model, goals showed no statistically significant association with high PFI in any age group. There were self-efficacy differences between the low- and high-fit men in the youngest group and differences in all the PFI categories in the middle-age and oldest groups. Moderate fitness was not related to better self-efficacy in the youngest group. The youngest men in the moderate and high PFI groups were likely to score well on social support, but no statistically significant differences between the PFI categories were found in the oldest group.

## Discussion

Results indicated that the majority of the low-fit men who participated in this study were health conscious and had intentions to increase their PA. However, there were age-group differences in self-rated PF and psychosocial factors. A greater proportion of the middle-aged (35–49 years) low-fit men self-reported poor fitness than either their younger (18–34 years) or older (50–64 years) counterparts. A half of the middle-aged low-fit men were inactive. Age differences were also found when psychosocial scores were compared with scores in the other PFI categories. Low fitness was related to lower scores in skills, goal setting and self-efficacy, regardless of age. However, knowledge was not related to high PFI in the youngest group and social support was not related to better PFI in the men aged 50–64.

### Self-estimated physical fitness

Previous studies have found that 50–60 per cent of the inactive population overestimate their PA [13,16,44]. In this study, almost 80 per cent of the young low-fit men reported moderate or good PF. A recent study reported that PA overestimators tended to compare their activity level to people who were even more sedentary than themselves [15]. Similarly, low-fitness men may use a downward comparison with more unfit people. However, in this study, the proportion of PF overestimators should be interpreted with caution. Self-rated fitness was compared with PFI, which has not been established as a measure of PF in previous studies. Thus, PFI may be fairly accurate measure for men aged 35–49, but underestimate PF in younger or older men.

The percentage of men aged 35–64, who self-estimated sufficient PA, was almost the same as the percentage who reported at least 3 hours PA per week. Although overestimation may be an obstacle to PA change [13,15,44], the present results suggest that most low-fit men have a realistic perception of their need to increase their PA. Nevertheless the possibility remains that, among the low-fit population, a sedentary lifestyle is the norm, blurring a clear perception of what constitutes sufficient PA or good PF [15]. Rhodes & Dean (2009) pointed out that inactivity is a heterogeneous phenomenon [45]. Sedentary behaviour may be as intentional as is participation in PA [45]. They suggest that rather than trying to advise sedentary people to increase their PA, it may be more fruitful to recommend them to plan how to cut down on sedentary time [45].

### Psychosocial factors

Good PA knowledge was related to moderate fitness in the youngest men. This may reflect different values and attitudes to PA among young men. Both sedentary behaviour and regular PA training may be habitual and related to the social environment. Berge et al. (2012) found that young men were more likely to engage in PA over 3.5 h/week, when their significant others had positive attitudes to healthy behaviours [46]. Social support was also higher in the youngest moderate and high-fit men than low-fit men. Moderately fit young men may have a greater tendency to cite health benefits and feeling refreshed as reasons for their engagement in PA than those who are either sedentary or athletic. The moderate-fit youngest men were also more likely to have set themselves exercise goals than their low-fit peers. This result indicates that moderately-fit young men invest effort in planning their engagement in PA, which also serves to underline the importance of social and environmental support in promoting PA.

Previous studies suggest that poor exercise skills may lead to negative experiences of PA, reduced self-efficacy and withdrawal from intended exercise activities [47]. Self- efficacy is the largest determinant to PA, and it is related both to the ability to overcome barriers and the confidence to engage in PA behaviour itself [26]. However, in the youngest low-fit age group, skills and self-efficacy tended to differentiate only the low- and high-fit, but not low- and moderate-fit men. Skills and self-efficacy are important long-term predictors for PA maintenance [26,48,49], and therefore genetics may have a stronger role in determining low PF than long-term PA in young adults. The likelihood of adopting PA habits increases if one has good motor skills for exercise activities or a genetic predisposition to good aerobic capacity and muscular strength [50,51]. The influence of PA history, overweight and chronic diseases broadens the gap between the fit and unfit during middle-age and the later working years [2,48].

Social support is a key factor for successful PA change [14]. However, social support was not related to better PFI in the oldest men. A recent review also concluded that social support is not a determinant of PA [21]. The present results suggest that social support appears to have more impact on PA in younger than older men. Lack of self-efficacy, motivation or PA skills may be more notable obstacles to engagement in PA in the later than earlier working years, and hence related to poor PA history. Previous research indicates that a positive social environment increases self-efficacy towards behaviour and mediates PA changes [12,16]. Social factors have been emphasized as an important component of PA programs for middle-aged men [12]. However, the specific form of social support should be targeted to low and moderately fit men differently.

Overall, the low-fit men were mostly overweight, perceived their exercise skills as poor, and were not very confident of their ability to achieve their exercise goals. A previous review concluded that intervention methods like action planning, self-monitoring and social support can increase self-efficacy and mediate PA change in a non-obese population [52]. However, in an obese population, effective intervention techniques may lie more in action itself. It has been suggested that planning does not promote PA if one’s confidence in actualizing the behaviour is low [47]. Compared to other health behaviour changes, regular PA requires more time and also, to some extent, special skills [23,26]. Good physical fitness may be related to one sort of physical activity capital that promotes engagement in PA and provides the ability to obtain social support from the environment [42]. While easy access to exercise groups and good sport facilities could be enough to increase PA in moderately-fit men, low-fit men may need more individual counseling, social support and PA alternatives that are perceived as agreeable and fun. Further research is needed to determine whether the PFI used here could form one component of a practical tool-kit in health counselling.

### Strengths and limitations

This study has limitations that restrict the generalizability of the findings. First, the participants were working-aged men who voluntarily engaged in the testing events. It is probable, therefore, that the study did not include men with the lowest fitness status. Unfit and inactive populations do not usually engage in PF studies owing to the challenging nature of the fitness tests used and lack of motivation [12]. However, the present data were obtained in public events that were free of charge and the fitness tests were easy to perform. Notwithstanding, the data were restricted to motivated men, as only 16 per cent of the participants were sedentary. Second, the study was cross-sectional in nature. Generation and age cohort differences in PA patterns and attitudes may influence the differences in the results for PF and the psychosocial factors.

Third, psychosocial factors were assessed by using constructs drawn from several health behaviour theories (e.g. the theory of planned behaviour and the Transtheorethical model) [22,25,26]. Rhodes & Nigg (2011) proposed that simply picking and choosing constructs from theories and models without making a full attempt to validate a theory may lead to problems in advancing PA research [26]. However, Glanz & Bishop suggested that the strongest interventions may be built from multiple theories [22]. The psychosocial measures used in this study had been validated or piloted in previous studies [40-43] among the target population, which strengthens the reliability of the results.

The fourth limitation concerns self-reported variables. For the psychosocial factors and physical activity, self-evaluations have had satisfactory validity [25,53]. Self-reported PF correlates moderately with objectively measured PF, although the most inactive groups tend to overestimate their PF [54]. It is recommended that other assessment methods be used together with self-reports [54]. This study included both evaluation of PF with objective fitness tests and self-rated PF. Physical fitness was used as an outcome measure instead of PA, because it has been suggested that PF may more accurately describe peoples’ general tendency to PA than self-reported PA [55]. However, chronic diseases or genetics may be the primary reasons for low PF, and not inactivity [3,21,50].

Assessing peoples’ PA in free-living conditions is challenging. Therefore, PF is a better predictor of health status than PA. PF was assessed in the present study with a PFI that comprised several dimensions of health-related fitness. The PFI did not indicate functional ability alone, but also risk for adverse health conditions. The PFI described PF (aerobic capacity and skeletal muscle strength) and indicated risk factors for functional disability and chronic diseases (fat% and VFA). However, this was the first time that this PFI has been used for research purposes. The reference values of the PFI tests were adjusted for the population of Finnish middle-aged men, which restricts its use in other populations. Further research should examine the validity of the present PFI in different age groups and on different fitness levels.

## Conclusions

Poor exercise skills, self-efficacy and social support were related to low PFI. Physical activity promotion for low-fit men should take into account age differences in the relationship between psychosocial factors and physical fitness. Thus, new and effective ways to establish social support and motivation for physical activity among low-fit men in all working-age groups are needed. Further research is also warranted on whether estimation of PFI could be used as a practical health counselling tool.

## Competing interests

The authors declare that they have no competing interests.

## Authors’ contributions

KSK analysed the data and wrote the manuscript. JK, KK and MP contributed to the study design, data collection and critical review of draft manuscripts. JV assisted with the statistical analysis, interpretation of data and critical review of draft manuscripts. All the authors read and approved the final manuscript.

## Pre-publication history

The pre-publication history for this paper can be accessed here:

http://www.biomedcentral.com/1471-2458/13/1113/prepub
